# Dynamic mRNA and miRNA expression of the head during early development in bighead carp (*Hypophthalmichthys nobilis*)

**DOI:** 10.1186/s12864-022-08387-x

**Published:** 2022-03-01

**Authors:** Weiwei Luo, Junru Wang, Ying Zhou, Meixia Pang, Xiaomu Yu, Jingou Tong

**Affiliations:** 1 State Key Laboratory of Freshwater Ecology and Biotechnology, Hubei Hongshan Laboratory, Institute of Hydrobiology, Innovation Academy of Seed Design, Chinese Academy of Sciences, Wuhan, 430072 China; 2 University of Chinese Academy of Sciences, Beijing, 100039 China; 3 Postdoctoral Innovation Practice Base, Shenzhen Polytechnic, Shenzhen, 518055 China

**Keywords:** Head development, *Hypophthalmichthys nobilis*, RNA–Seq, sRNA–Seq

## Abstract

**Background:**

Head of fish species, an exquisitely complex anatomical system, is important not only for studying fish evolution and development, but also for economic values. Currently, although some studies have been made on fish growth and body shapes, very limited information is available on the molecular mechanism of head development.

**Results:**

In this study, RNA sequencing (RNA–Seq) and small RNA sequencing (sRNA–Seq) technologies were used to conduct integrated analysis for the head of bighead carp at different development stages, including 1, 3, 5, 15 and 30 Dph (days post hatch). By RNA-Seq data, 26 pathways related to growth and bone formation were identified as the main physiological processes during early development. Coupling this to sRNA–Seq data, we picked out six key pathways that may be responsible for head development, namely ECM receptor interaction, TNF signaling pathway, osteoclast differentiation, PI3K–Akt signaling pathway, Neuroactive ligand–receptor interaction and Jak–STAT signaling pathway. Totally, 114 important candidate genes from the six pathways were obtained. Then we found the top 20 key genes according to the degree value by cytohubba, which regulated cell growth, skeletal formation and blood homeostasis, such as *pik3ca*, *pik3r1*, *egfr*, *vegfa*, *igf1* and *itga2b*. Finally, we also acquired 19 key miRNAs playing multiple roles in the perfection of various tissues in the head (such as brain, eye and mouth) and mineralization of head bone system, such as let–7e, miR–142a–5p, miR–144–3p, miR–23a–3p and miR–223.

**Conclusions:**

Results of this study will be informative for genetic mechanisms of head development and also provide potential candidate targets for the interaction regulation during early growth in bighead carp.

**Supplementary Information:**

The online version contains supplementary material available at 10.1186/s12864-022-08387-x.

## Introduction

The head of vertebrate is an extremely complex organ, which is one of the classic research topics of evolutionary morphology and developmental biology [1]. Species evolved into different head shapes and sizes (skull morphologies) to adapt to their living environment [2]. The evolutionary morphology and developmental biology of head have been reported in a variety of animals, such as dogs [3, 4], bird [5, 6], and ruminant [7, 8]. Head shapes and sizes of fish are very important not only for the studies on fish evolution and development, but also for the economic value of aquaculture industry, which directly affect the fillet yield of economic fish [2]. For most aquaculture fish, smaller head and uniform body shape provide a higher percentage of meat yield. Therefore, selecting smaller head and streamlined body shape is of great significance in aquaculture [9]. Because skull morphology of fish is one of the commercial traits, it is very important to study the genetic regulation of head development. The ultimate goal of researchers is to produce fish with very large or very small head for ornamental or economic value [10]. Genome-wide association study (GWAS) and quantitative trait loci (QTL) mapping of head shapes and sizes were performed in fish species, including common carp [10, 11], German mirror carp [12] and catfish [2]. Although some studies have been made on the genetic architecture of the shape and size of fish head, little is known about the genetic control basis of the head development in aquaculture fish.

Bighead carp (*Hypophthalmichthys nobilis*) is one of the most important cultured fishes in Asia, which provides a large amount of high-quality protein for human beings [13]. Principally produced in China, the global production of bighead carp reached 3.14 million tons in 2018 (FAO). For most aquaculture fish, smaller head and uniform body shape provide a higher percentage of meat yield [9]. Unlike other fish, the head of bighead carp is a raw material of many traditional Chinese dishes (Steamed Fish Head with Diced Hot Red Peppers, and so on), and the head is more popular than its fillet with even higher economic value. In addition, the nutrients of brain pulp, phospholipid and the soft skin with collagen in the head of bighead carp are rich, and therefore the head has higher nutritional value [14]. To meet the market demand for bighead carp, selection for fast–growing and big–headed varieties is prevalent among current breeding programs. At present, although some researchers have made some progresses on the genetic basis of fish growth and body shape, there are few reports on the molecular mechanisms of head development.

MicroRNAs (miRNAs) are small, endogenous and noncoding RNAs, about 22 nucleotides long; MiRNAs regulate gene silencing through partial messenger RNA (mRNA) excision and translation inhibition by binding to target mRNA [15]. Generally, a single miRNA regulates multiple target genes, and a single gene is also regulated by multiple miRNAs, so the interaction between miRNAs and target genes is complicated [16]. MiRNAs play important roles in regulation and control of growth, development, immunity and reproduction [17–20]. Benefiting from the advancement of sequencing technology, next-generation sequencing technology (NGS) provides powerful tools for studies on fish life activities and environmental adaptation, such as RNA sequencing (RNA-Seq) and microRNA sequencing (sRNA-Seq) [21]. Currently, the integrated analysis of mRNA and miRNA expression profiles has been performed on several fish species, including zebrafish [19, 22], blunt snout bream [18], Japanese flounders [21, 23], largemouth bass [24] and yellowfin seabream [20].

In the present study, we used NGST to conduct the RNA-Seq and sRNA-Seq from the head of bighead carp at different development stages, including 1Dph (days post hatch) group, 3Dph group, 5Dph group, 15Dph group and 30Dph group. This is the first report on integrated analysis of RNA-Seq and sRNA-Seq in bighead carp and as such offers deeper insight into the genetic mechanisms of head development. Furthermore, we identified a set of differentially expressed unigenes (DEGs) and differentially expressed miRNA (DEmiRs) from early growth, bone formation and blood homeostasis signaling pathways, which potentially services as valuable resources for future molecular breeding studies on head-related traits in fish species.

## Results

### Sample characterization

**Fig. 1:**
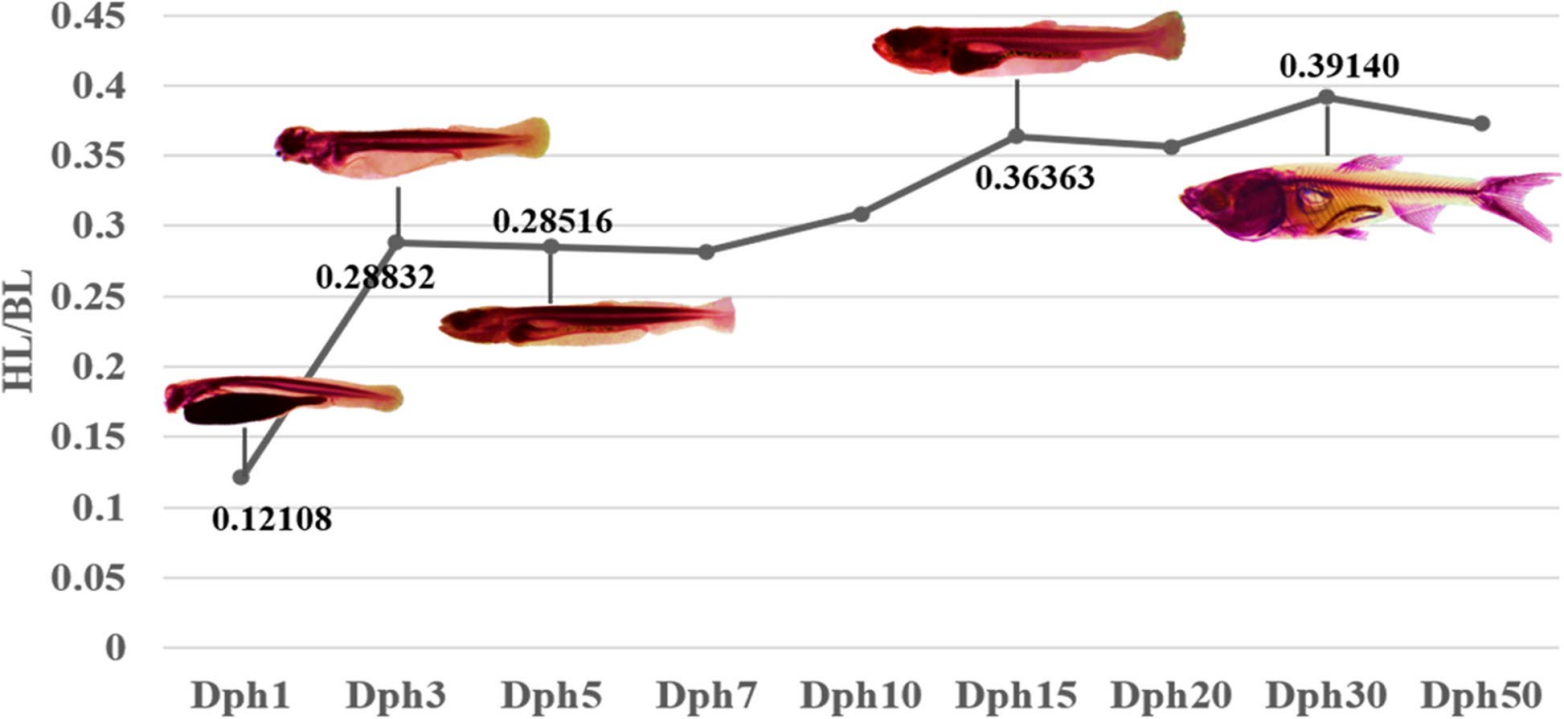
The ratios of head length and body length of bighead carp during early developmental stages. Dph: days post hatch; BL: body length; HL: head length; HL/BL: the ratios of HL and BL. HL/BL of Dph1 was the ratio of HL/TL

To identify cues controlling molecular pathways associated with head development and skull morphology in bighead carp, we characterized differentially expressed mRNAs and miRNAs of the head in bighead carp samples from different development stages. To select suitable head tissue samples, the total length (TL), body length (BL), and head length (HL) of 15 bighead carp individuals in each development stage, including 1Dph (days post hatch) group, 3Dph group, 5Dph group, 7Dph group, 10Dph group, 15Dph group, 20Dph group, 30Dph group and 50Dph group, were measured in this study (Table 1). The ratios of HL and BL (HL/BL) from these different development stages were calculated simultaneously. The HL/BL value showed an initial increase from 1Dph to 3Dph, then slowly increased from 3Dph to 15Dph, reached the peaking at Dph30 (Fig. 1). During the development stages from 3Dph to 5Dph, the HL/BL values were similar while the morphological characterizations changed greatly, such as the transformation of food habit from endogenous to exogenous, the progressive development of feeding and digestive system and the accumulation of pigment cells. Based on morphological characterizations and the HL/BL values (Fig. 1), the whole head in the samples of five different developmental stages (1Dph, 3Dph, 5Dph, 15Dph and 30Dph) were selected for the integrated analysis of RNA-seq and sRNA-Seq in bighead carp.

**Table 1:** Sample characterization of bighead carp during early developmental stages

Item	TL (mm)	BL (mm)	HL (mm)	HL/BL
Dph1	6.85 ± 0.23		0.83 ± 0.07	0.12 ± 0.01^a^
Dph3	7.51 ± 0.51	6.33 ± 0.43	1.82 ± 0.16	0.29 ± 0.03^b^
Dph5	8.95 ± 0.33	7.70 ± 0.41	2.20 ± 0.21	0.29 ± 0.02^b^
Dph7	9.35 ± 0.45	8.14 ± 0.46	2.30 ± 0.31	0.28 ± 0.02^b^
Dph10	10.49 ± 1.29	9.23 ± 1.26	2.87 ± 0.65	0.31 ± 0.03^b^
Dph15	12.25 ± 0.97	10.41 ± 0.91	3.77 ± 0.20	0.36 ± 0.02^c^
Dph20	19.89 ± 3.02	15.70 ± 2.36	5.61 ± 0.99	0.36 ± 0.02^c^
Dph30	21.56 ± 2.28	16.49 ± 1.60	6.44 ± 0.50	0.39 ± 0.02^c^
Dph50	32.34 ± 3.67	24.91 ± 2.76	9.28 ± 1.22	0.37 ± 0.03^c^

*Dph* days post hatch, *TL* total length, *BL* body length, *HL* head length. HL/BL: the ratios of HL and BL. HL/BL of Dph1 was the ratio of HL/TL. Different lowercase letters meant significant differences of HL/BL among different development stages (*P* < 0.05)

### Global analysis of head transcriptome during different developmental stages

15 Illumina RNA-Seq libraries constructed from the whole head in the samples of five different developmental stages (1Dph, 3Dph, 5Dph, 15Dph and 30Dph) of bighead carp were sequenced. After trimming process and filtering out the low-quality reads, a total of 136.97 G clean reads were produced from all samples. Totally, 27.53 Gb, 26.85 Gb, 26.60 Gb, 32.65 Gb and 23.34 Gb of clean bases were generated in 1Dph group, 3Dph group, 5Dph group, 15Dph group and 30Dph group, respectively. The Q30 value of each sample was up to 94.35%, and the GC distribution of each sample ranged from 45.83 to 46.66% (Table S1). With the Trinity *de novo* assembler, a total of 360,144 transcripts were generated, with an N50 of 2894 bp. A total of 162,698 unigenes were further generated with an N50 of 1749 bp. The size distribution of these unigenes was shown in Fig. S1. A total of 143,591 unigenes were successfully annotated through alignment to reference databases. In total, 36,086 (22.17%), 134,161 (82.46%), 20,695 (12.71%), 33,510 (20.59%), 42,701 (26.24%), 42,701 (26.24%) and 15,710 (9.65%) unigenes could be annotated by NR, NT, KO, Swiss-Prot, PFAM, GO and KOG database, respectively, with 143,591 (88.25%) unigenes annotated in at least one Database. Annotation information of uingenes from RNA-Seq was shown in Table S2.

**Fig. 2:**
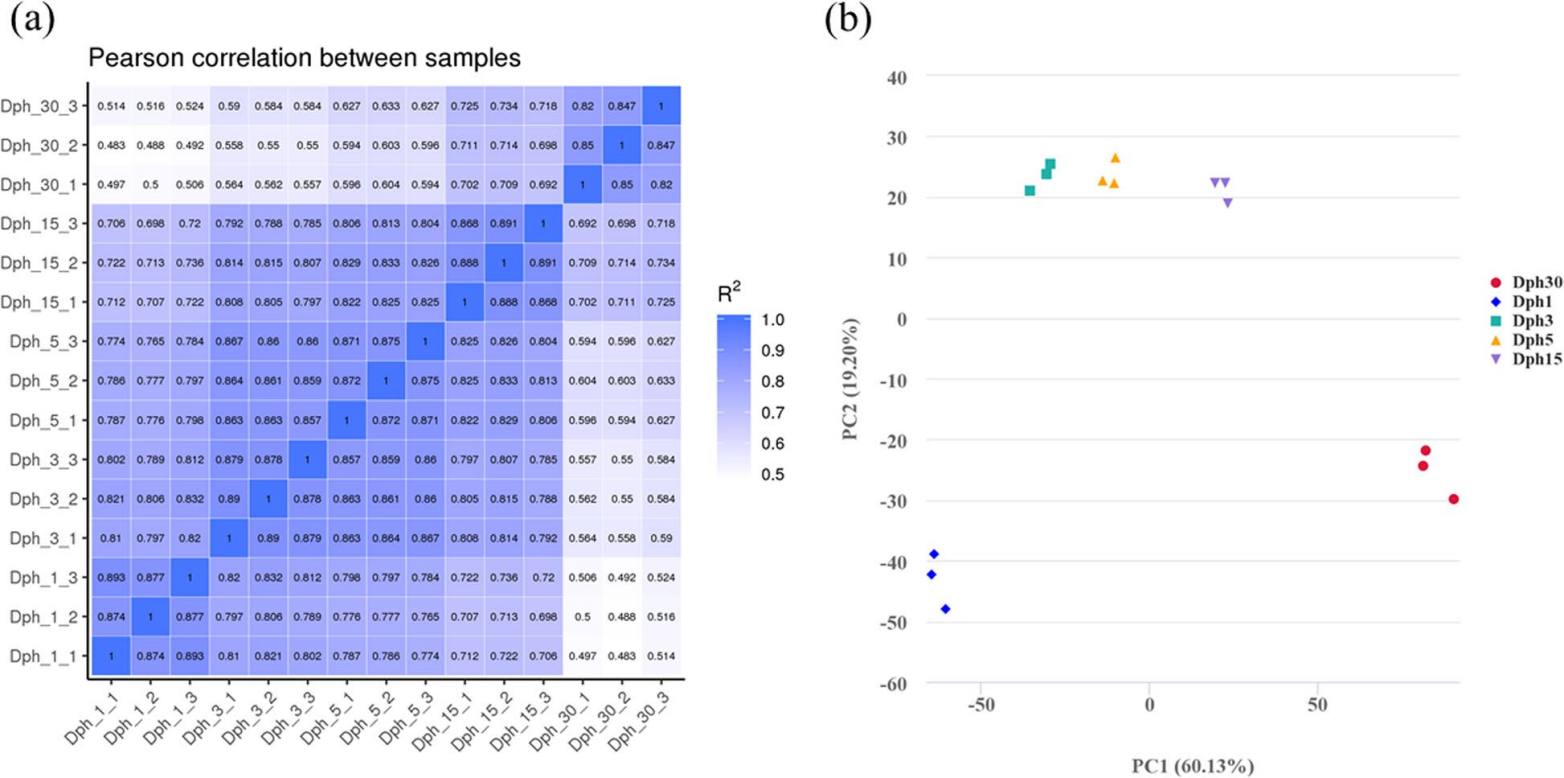
The repeatability of samples from five time points. **a** Correlation analysis of samples. The color of cross square represents the correlation degree between samples. **b** PCA analysis of transcriptome differences among five groups

Based on the annotation, hundreds of genes and KEGG pathways related to head development and skull morphology were identified in the head transcriptome (Table 2), such as TGF-β signaling pathway (*tgfb1*), Insulin-like growth factors (Igf) signaling pathway (*igf1*) and BMP pathway (*bmp 3*). A correlation analysis and principal component analysis (PCA) were performed on these 15 samples and the repeatability of samples from five time points was acceptable (Fig. 2).

**Table 2:** Main regulatory pathways of head development in bighead carp

Number	Pathway Name	Number	Pathway Name
1	AMPK signaling pathway	14	NF–kappa B signaling pathway
2	Calcium signaling pathway	15	Notch signaling pathway
3	cAMP signaling pathway	16	Osteoclast differentiation
4	cGMP–PKG signaling pathway	17	PI3K–Akt signaling pathway
5	ECM–receptor interaction	18	PPAR signaling pathway
6	ErbB signaling pathway	19	Rap1 signaling pathway
7	Estrogen signaling pathway	20	Ras signaling pathway
8	FoxO signaling pathway	21	Regulation of actin cytoskeleton
9	Hedgehog signaling pathway	22	TGF–beta signaling pathway
10	Insulin signaling pathway	23	Thyroid hormone signaling pathway
11	Jak–STAT signaling pathway	24	TNF signaling pathway
12	MAPK signaling pathway	25	VEGF signaling pathway
13	Neuroactive ligand–receptor interaction	26	Wnt signaling pathway

### Identification and function analysis of DEGs of head transcriptome during different developmental stages

**Fig. 3:**
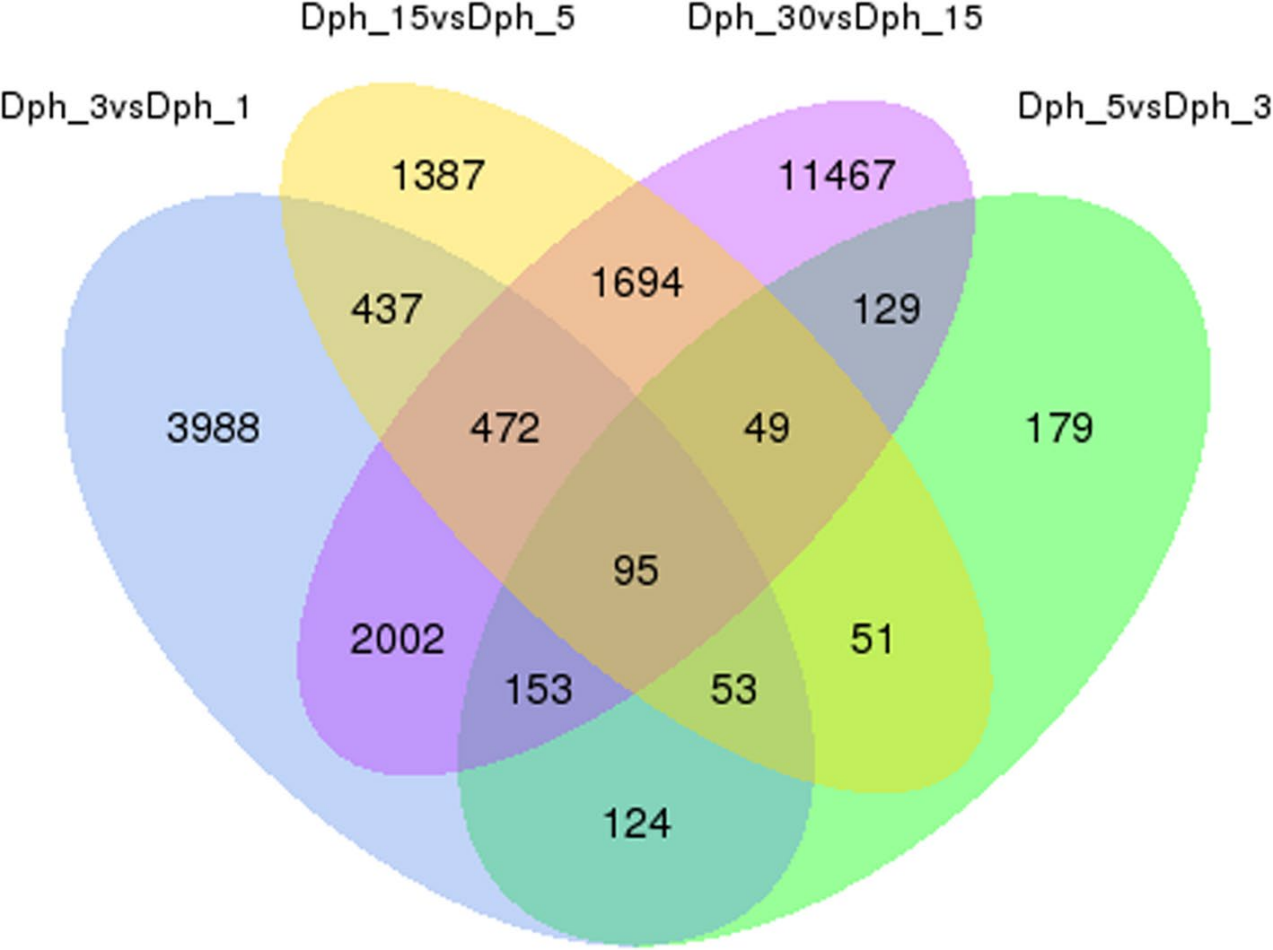
The DEG numbers of bighead carp in comparison groups of adjacent development stages

Based on the criteria of |fold-change| ≥2 and FDR ≤ 0.01, the differentially expressed unigenes (DEGs) were identified of head transcriptome during different developmental stages. Comparison of adjacent development stages, Dph3vsDph1, Dph5vsDph3, Dph15vsDph5 and Dph30vsDph15 detected 7324, 833, 4238 and 16,061 DEGs, with 95 overlapping DEGs detected in all the four comparison groups (Fig. 3). Besides the comparison of adjacent development stages, we also compared the DEGs in nonadjacent development stages. Dph5 vs Dph1, Dph15 vs Dph1, Dph30 vs Dph1, Dph15 vs Dph3, Dph30 vs Dph3 and Dph30 vs Dph5 identified 9703, 15,945, 28,055, 7024, 25,314 and 22,919 DEGs, respectively. The highest number of DEGs was observed in Dph30 vs Dph1 comparison group, which suggested significantly morphological shifts from Dph1 to Dph30. These extensive differences of DEGs in adjacent and nonadjacent development stages had close relationship with the morphological and physiological variation, which further proved that selected 5 developmental stages was typical morphological period during head development of bighead carp.

### General miRNA Analysis of head during different developmental stages

A total of 22.8 M, 20.5 M, 23.1 M, 20.9 M, and 20.1 M raw reads were collected from the whole head in samples of five different developmental stages (1Dph, 3Dph, 5Dph, 15Dph and 30Dph) of bighead carp, respectively. After discarding low-quality reads, 21.7 M, 20.2 M, 22.8 M, 20.7 M, and 19.7 M clean small RNA reads were obtained in 1Dph group, 3Dph group, 5Dph group, 15Dph group and 30Dph group, respectively. In total, 949 miRNAs were identified, of which 249 were known miRNAs and 700 were novel miRNAs.

**Fig. 4:**
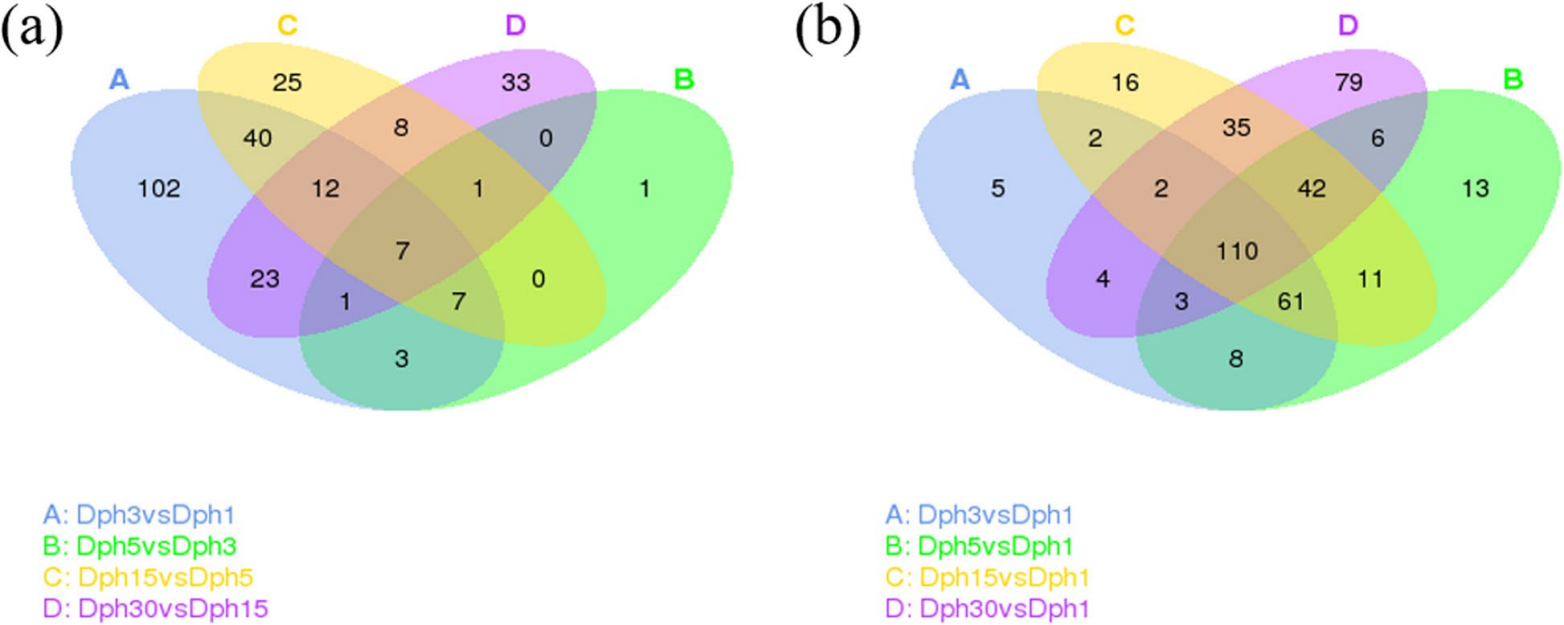
Detection of homologous miRNAs in the head of bighead carp among different comparison groups. **a** The DEmiR numbers at adjacent development stages. **b** The DEmiR numbers at nonadjacent development stages

Based on a corrected P value (p-adjust/padj) < 0.05, we identified 195 (112 up-regulated and 83 down-regulated), 20 (20 up-regulated and 0 down-regulated), 100 (68 up-regulated and 32 down-regulated) and 85 (35 up-regulated and 50 down-regulated) differentially expressed miRNAs in adjacent pairwise comparisons of Dph3 vs Dph1, Dph5 vs Dph3, Dph15 vs Dph5 and Dph30 vs Dph15. Compared with these adjacent pairwise comparisons, nonadjacent pairwise comparisons obtained more differentially expressed miRNAs (Table 3). Through Venn diagram analysis, 7 and 110 overlapped differentially expressed miRNAs were identified in the successive development stages and the following four development stages using Dph1 as reference stage, respectively (Fig. 4).

**Table 3:** Number of differentially expressed miRNA in head of bighead carp among different developmental stages

Group	Up–expressed	Down–expressed	Differential–expressed
Dph3 vs Dph1	112	83	195
Dph5 vs Dph3	20	0	20
Dph15 vs Dph5	68	32	100
Dph30 vs Dph15	35	50	85
Dph5 vs Dph1	145	109	254
Dph15 vs Dph1	158	121	279
Dph30 vs Dph1	130	151	281
Dph15 vs Dph3	72	31	103
Dph30 vs Dph3	107	102	209
Dph30 vs Dph5	102	124	226

### Analysis of miRNA-mRNA integration at five developmental stages

From the first day to the 30th day after hatching, significant morphological changes have taken place in the head of bighead carp, including the continuous perfection of various tissues in the head (such as brain, eye, mouth and other tissues) and the mineralization of the head skeletal system (brain skeleton and pharyngeal bone). Head development of bighead carp involves many vital movements, such as cell growth and death, organ development (such as immune system), signal transduction and nutrition metabolism (such as carbon metabolism), which indicates the complexity of head development of bighead carp.

**Fig. 5:**
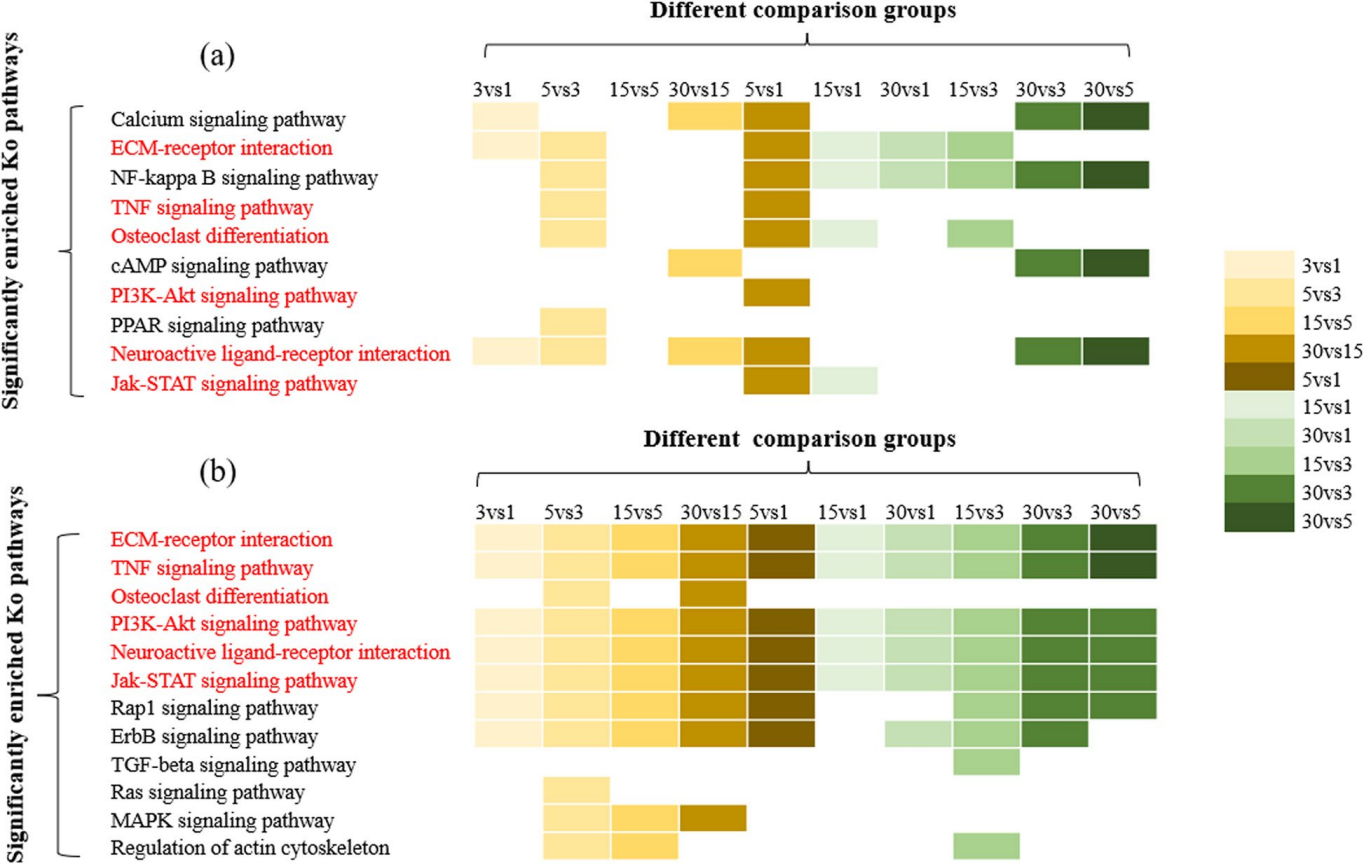
The significantly enriched KO pathways. **a** Pathways of differentially–expressed genes. **b** Pathways of target genes from differentially–expressed miRNAs. The color legend just meant different comparison groups. Common pathways were marked red color

**Fig. 6:**
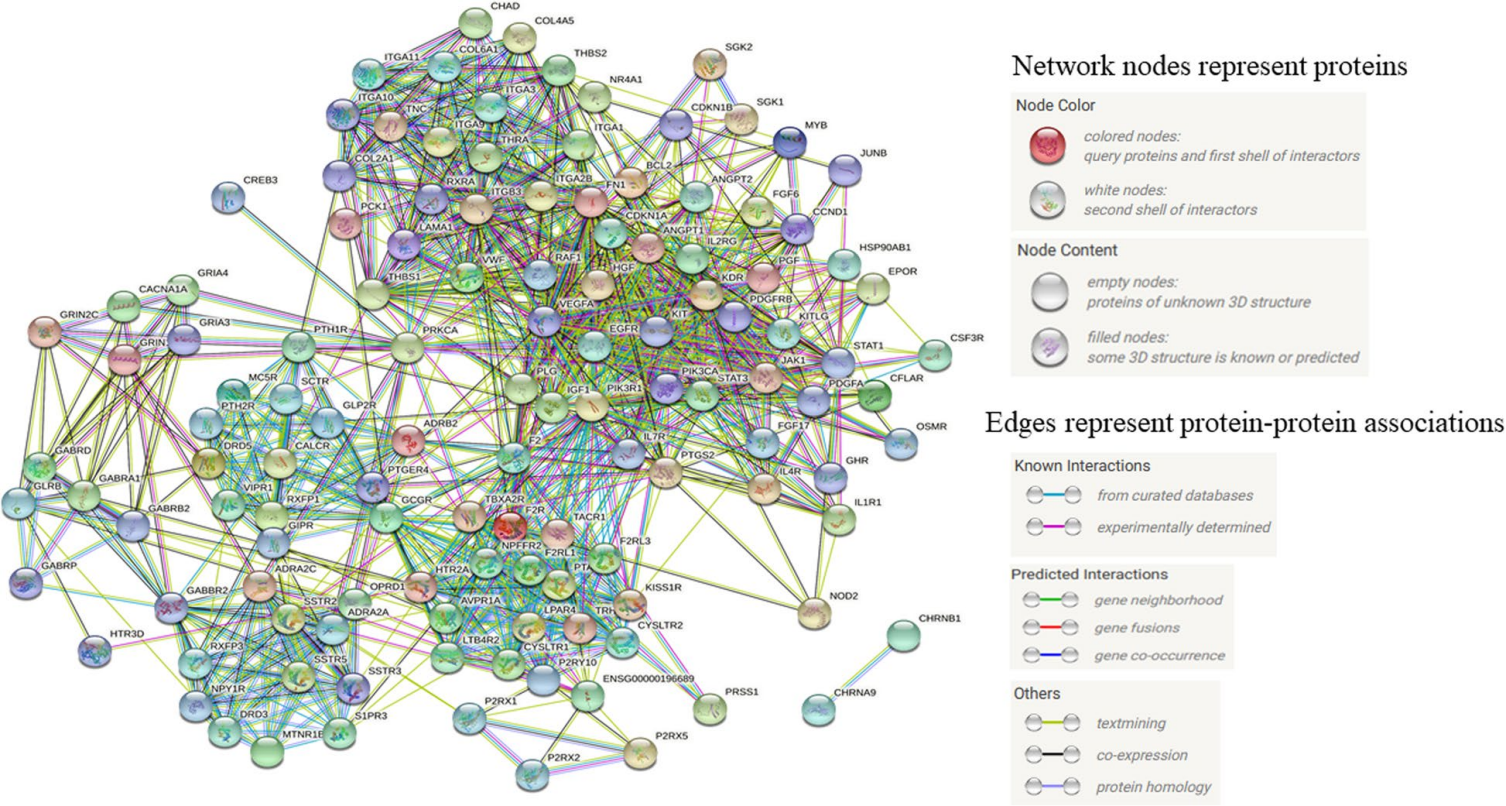
Protein–protein interaction network for the important candidate genes of head development in bighead carp. Different dots represent different enzymes, and different colored lines represent the interaction between proteins

**Fig. 7:**
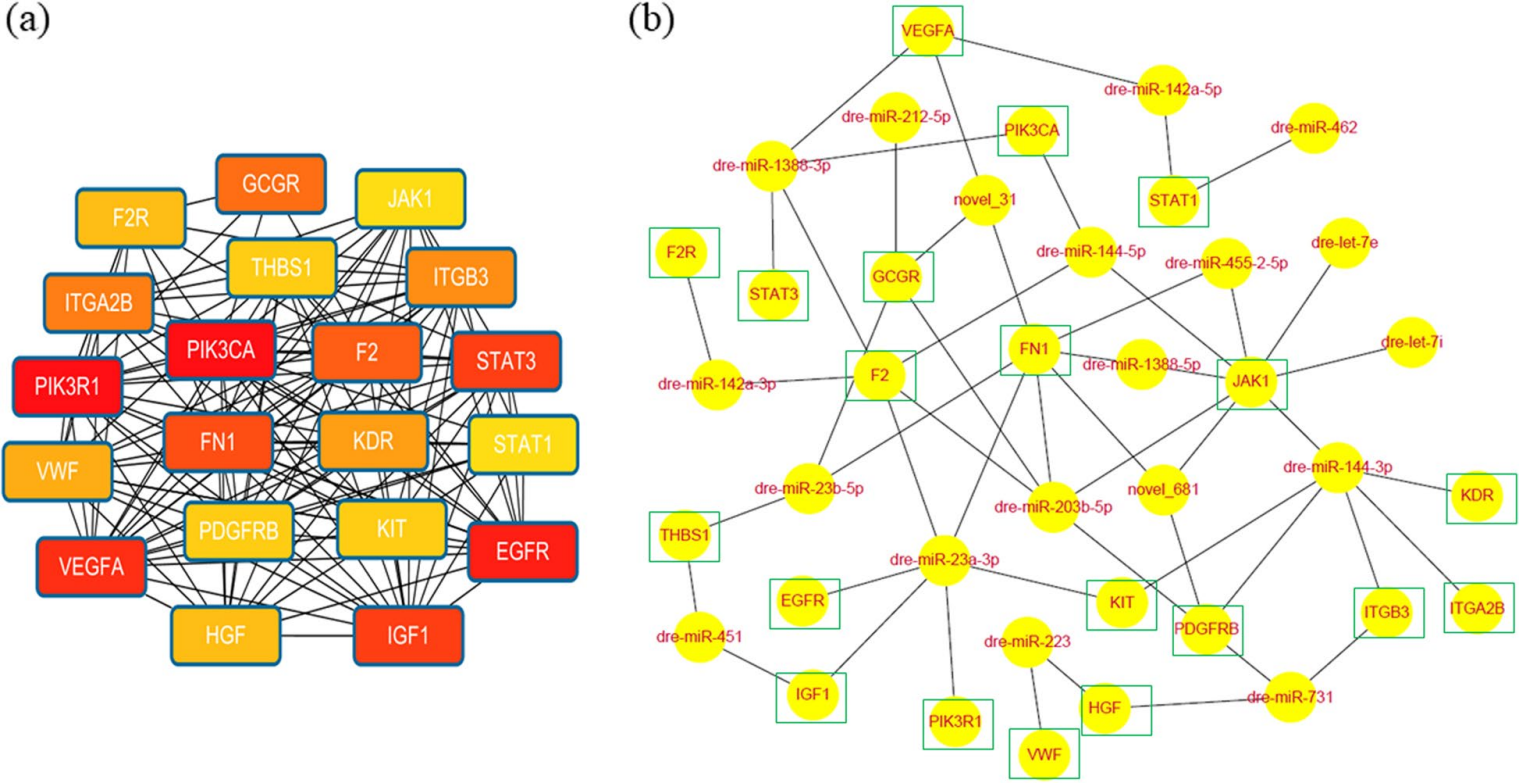
The interaction network of genes and miRNAs of head development in bighead carp. **a** The interaction network for the top 20 key genes by Cytohubba. Different nodes meant different genes. The color depth of nodes was determined by the degree value. The higher the rank was, the darker the color was. **b** The miRNA-mRNA interaction network for key genes and miRNAs. Green rectangular boxes meant key genes. The lines between miRNA and mRNA meant the interactions between them. Gene abbreviations: Epidermal growth factor receptor (*EGFR*), Prothrombin (*F2*), Proteinase-activated receptor 1 (*F2R*), Fibronectin type III domain containing (*FN1*), Glucagon receptor (*GCGR*), Hepatocyte growth factor (*HGF*), Insulin-like growth factor I (*IGF1*), Integrin alpha-IIb (*ITGA2B*), Integrin beta-3 (*ITGB3*), Tyrosine-protein kinase JAK1 (*JAK1*), Vascular endothelial growth factor receptor 2 (*KDR*), Mast/stem cell growth factor receptor Kit (*KIT*), Platelet-derived growth factor receptor beta (*PDGFRB*), Phosphatidylinositol 4,5-bisphosphate 3-kinase catalytic subunit alpha isoform (*PIK3CA*), Phosphatidylinositol 3-kinase regulatory subunit alpha (*PIK3R1*), Signal transducer and activator of transcription 1-alpha/beta (*STAT1*), Signal transducer and activator of transcription 3 (*STAT3*), Thrombospondin-1 (*THBS1*), Vascular endothelial growth factor A (*VEGFA*), Von Willebrand factor (*VWF*)

In the significant enrichment pathways of different comparison groups, 10 pathways and 12 pathways overlapped with the above 26 pathways (Table 2) from transcriptome and small RNA data were shown in Fig. 5a and Fig. 5b, respectively. The common six pathways enriched by differential genes and target genes of differential miRNA (Fig. 5) were ECM–receptor interaction, TNF signaling pathway, osteoclast differentiation, PI3K–Akt signaling pathway, neuroactive ligand receptor interaction and JAK–STAT signaling pathway. These six pathways were recognized as the key pathways of head development. A total of common 114 gene were obtained from differential genes and target genes of differential miRNA in the key pathways. Using string online database, the protein interaction map of these genes was drawn (Fig. 6). Based on the protein interaction map, the top 20 key genes (hub genes of 114 candidate genes) were found according to the degree value by cytohubba, which regulated cell growth, skeletal formation and blood homeostasis (Fig. 7a). The heatmap of key genes was shown in Fig. S2. Finally, we constructed the miRNA-mRNA interaction network based on the 19 differential miRNAs whose target genes were the top 20 key genes using Cytoscape software (Fig. 7b).

### Quantitative analysis of miRNA and mRNA expression

To validate the RNA-Seq and sRNA-Seq results, qRT-PCR was performed to detect the expression of 6 randomly selected differentially expressed mRNAs and 6 miRNAs. As shown in Fig. S3, the results of qRT-PCR were consistent with those of RNA-Seq and sRNA-Seq at 1Dph, 3Dph, 5Dph, 15Dph and 30Dph, respectively (Fig. S3).

## Discussion

Unlike other intensively-cultured fish, the head of bighead carp is a raw material of many traditional Chinese dishes, such as fish head with minced peppers, fish head hot pot, casserole fish head soup and fish head tofu soup. Therefore, the head is more valued than its fish meat [14]. To explore the molecular mechanisms of head development, we examined the RNA-Seq and sRNA-Seq data of bighead carp at different development stages, including 1Dph, 3Dph, 5Dph, 15Dph and 30Dph. Firstly, 26 pathways were identified as important pathways during early stages in the RNA-Seq data based on previous studies, which were closely related to growth development and bone formation [10, 18, 25, 26]. Then the common six pathways enriched by differential genes and target genes of differential miRNA were obtained. The top 20 key genes were found from the 114 candidate genes in the six key pathways. Finally, we also acquired 19 key miRNAs playing multiple roles in the perfection of various tissues in the head (such as brain, eye and mouth) and mineralization of head bone system.

### Key pathways regulating head development

The common six pathways enriched by differential genes and target genes of differential miRNA (Fig. 5) were ECM–receptor interaction, TNF signaling pathway, osteoclast differentiation, PI3K–Akt signaling pathway, neuroactive ligand receptor interaction and JAK–STAT signaling pathway. These six pathways were identified as the key pathways of head development in bighead carp. Wu et al. (2020) studied the transcriptome of breast muscle of Jinghai yellow chicken with different growth rate at early growth stage, and found that ECM – receptor interaction pathway was one of the enrichment pathways of differentially expressed genes, indicating that this pathway may play an important role in the growth and development of chicken [27]. As an important cytokine, tumor necrosis factor (TNF) can induce apoptosis, cell survival, inflammation and immunity. Wang and Guo (2021) studied the effects of TNF – α on the autophagy and NF – kappaB signaling pathway of fibroblast like synoviocytes in rheumatoid arthritis. The results showed that inhibition of autophagy can improve the imbalance of proliferation / apoptosis of fibroblast like synoviocytes aggravated by TNF – α to a certain extent, thus delaying the degree of rheumatoid arthritis [28]. In addition, Hurem et al. (2017) also found TNF – α took part in regulation of γ radiation on the early development of zebrafish [29]. Osteoclasts are the multi-nuclear red cells from the mononuclear macrophage line, responsible for bone absorption [30], and participate in the development and regeneration of organs. PI3K - Akt signaling pathway is activated by a variety of cellular stimuli or toxic damage, and regulates basic cellular functions such as transcription, translation, proliferation, growth and survival. TNF signaling pathway, osteoclast differentiation and PI3K – Akt signaling pathway are involved in the formation of intermuscular bone in blunt snout bream, and play important roles in bone formation and development [25].

Similar to this study, neuroactive ligand receptor interaction was also found in the development of chicken crowns [31] and biomineralization and shell formation of pearl oyster Pinctada [32], indicating that this pathway plays an important role in animal development and biomineralization. JAK–STAT signaling pathway is one of the few pleiotropic cascade pathways, which is used for development and steady-state signal transduction in a variety of animals from human to fly [33]. In mammals, JAK–STAT signaling pathway is the main signaling mechanism of a series of cytokines and growth factors. Therefore, these six pathways play important roles in the growth, development and bone formation of animals. They may be involved in the continuous perfection of various tissues in the head (such as brain, eye, mouth and other tissues) and the mineralization of head bone system (cranial skeleton and pharyngeal bone).

### Key genes regulating head development

The top 20 key genes (Fig. 7a) regulating cell growth, skeletal development and blood homeostasis were obtained from the 114 important candidate genes: phosphatidylinositol-4,5-bisphosphate 3-kinase catalytic subunit alpha (*pik3ca*), phosphoinositide-3-kinase regulatory subunit 1 (*pik3r1*), epidermal growth factor receptor (*egfr*), vascular endothelial growth factor A (*vegfa*), signal transducer and activator of transcription 3 (*stat3*), insulin like growth factor 1 (*igf1*), fibronectin 1 (*fn1*), coagulation factor II, thrombin (*f2*), glucagon receptor (*gcgr*), integrin alpha-IIb (*itga2b*), integrin beta-3 (*itgb3*), kinase insert domain receptor (kdr), von willebrand factor (*vwf*), *hepatocyte growth factor* (*hgf*), coagulation factor II thrombin receptor (*f2r*), thrombospondin 1 (*thbs1*), platelet derived growth factor receptor beta (*pdgfrb*), mast/stem cell growth factor receptor kit (*kit*), janus kinase 1 (*jak1*) and signal transducer and activator of transcription 1 (*stat1*).

Phosphatidylinositol-4,5-bisphosphate 3-kinase catalytic subunit alpha (PIK3CA) and phosphoinositide-3-kinase regulatory subunit 1(PIK3R1) are members of PI 3-Kinases [34, 35], which are involved in many cellular functions, including growth factor receptor signaling, cytoskeletal organization, apoptosis and so on [36]. Epidermal growth factor receptor (EGFR) is a kind of cell surface protein binding epidermal growth factor, which can promote cell proliferation by inducing receptor dimerization and tyrosine autophosphorylation [37]. Insulin like growth factor 1 (*igf1*) and hepatocyte growth factor (*hgf*) are closely associated with growth and development, which play important roles in cell growth, cell motility and morphogenesis [38, 39]. Studies on *igf1* gene have shown that its mutation can affect the growth and development and reduce body size and weight of animal, such as human, dog and mouse [3, 40, 41]. Mast/stem cell growth factor receptor kit (KIT) acts as a cell surface receptor. It regulates many essential biological processes, including cell survival and proliferation, hematopoiesis, stem cell maintenance and melanogenesis [42]. These kinases, growth factors and growth factor receptors may play important roles in the head development of bighead carp by regulating cell proliferation, cell growth, cell motility and morphogenesis.

Fibronectin 1 (FN1) is capable of binding cell surface and various compounds, such as fibrin, collagen, actin, DNA and heparin. *Fn1* regulates cell adhesion, cell movement, wound healing and so on [43]. Moreover, *fn1* plays an important role in the process of osteoblast compaction, which is necessary for osteoblasts mineralization [44]. Integrin alpha-IIb/beta-3 (ITGA2B:ITGB3) acts as a receptor for multiple proteins, such as fibronectin, prothrombin, and thrombospondin. It is involved in chondrocyte growth and proliferation [45]. Signal transducer and activator of transcription 3 (STAT3) protein changes the expression level of various genes to adapt to cell stimuli from many cytokines and growth factors, including HGF, EGF, BMP2 and IL5 [46]. It has been reported *stat3* gene plays a key part in cell growth and apoptosis [47]. *Stat1* gene is also identified in the head development of bighead carp, which is an important paralog of *stat3*. Studies on thrombospondin 1 (THBS1) protein have shown that THBS1 plays an important role in mediating cell-to-cell and cell-to-matrix interactions [48]. Nie et al. (2019) found *stat1*, *thbs1* and thrombospondin at the early stage of intermuscular bone development, believing that they play an active role in osteoblast differentiation and intermuscular bone formation [25]. The identification of genes regulating bone formation suggested that development and mineralization of bone system coordinated with head development of bighead carp.

Vascular endothelial growth factor A (*vegfa*) encodes a heparin-binding protein, which exists as a disulfide-linked homodimer [49]. Kinase insert domain receptor (KDR), known as vascular endothelial growth factor receptor 2, acts as a cell-surface receptor for VEGFA, VEGFC and VEGFD [50]. *Vegfa* and *kdr* play essential roles in the regulation of angiogenesis, vascular development, vascular permeability, and embryonic hematopoiesis [51]. In this study, we also identified coagulation Factor II, thrombin (*f2*) [52], coagulation factor II thrombin receptor (*f2r*) [53], von willebrand factor (*vwf*) [54], janus kinase 1(*jak1*) [55] and glucagon receptor (*gcgr*) [56], which function in blood homeostasis, inflammation and wound healing. Platelet derived growth factor receptor beta (*pdgfrb*) is essential for normal development of the cardiovascular system and rearrangement of the actin cytoskeleton, and has roles in the regulation of many biological processes including embryonic development, angiogenesis, cell proliferation and differentiation [57]. Angiogenesis, vascular development, blood homeostasis and inflammation are involved in the tissue/organ development of animals. These genes associated with angiogenesis may regulate head development of bighead carp, providing blood homeostasis for the perfection of various tissues (brain, muscles, lipids, etc.) and the development of head bone system.

### Key miRNAs regulating head development

Finally, we also acquired 19 key miRNAs (Fig. 7b) playing multiple roles in the regulation of cellular proliferation and differentiation, metabolism, immunity, regeneration and many others. let-7e and let-7i belonging to the let-7 family demonstrated to be associated with the development of cancer [58, 59]. Hu et al. reported that let-7i was highly correlated with physiological and growth characteristics of velvet antlers [60]. MiR-223 is a hematopoietic cell-derived miRNA that plays multiple roles in regulation of monocyte-macrophage differentiation, neutrophil recruitment, innate immunity, cancer and optic nerve regeneration [22, 61]. Xie et al. have reviewed miR-223 exerts various effects on bone metabolism, especially in the processes of osteoclast and osteoblasts differentiation and highlighted the potential clinical applications of miR-233 in bone metabolism disorders [62].

miR-23a-3p and miR-23b-5p were recognized as candidate miRNAs regulating gene expression related to physiological responses to hypoxia in the liver of largemouth bass [63]. miR-23a-3p played roles in osteoblast proliferation and differentiation [64], cartilage regeneration [65] and inflammatory response [66]. microRNA-23b-5p regulated cell proliferation of hepatocellular carcinoma [67] and brown adipogenesis and thermogenic program in enhanced brown adipose tissue [68]. MiR-142a-3p was involved in the inflammatory response in grass carp [69], cute hypoxic stress in largemouth bass [63] and vascular development and integrity in zebrafish [70]. miR-142a-5p played critical roles in inflammation and tumorigenesis. Yuan et al. (2020) reported that miR-142a-5p promoted osteoblast differentiation via targeting nuclear factor IA [68].

Some studies have confirmed that the teleost-specific miR-462/731 cluster was involved in some biological processes, such as the immune responses to viral pathogens in Atlantic Salmon [71] and Atlantic cod [72], hypoxia [73] in zebrafish and blunt snout bream [74]. Huang et al. (2020) found significant reduction of digestive organs, especially liver and exocrine pancreas after the miR-462/miR-731 knockdown during zebrafish liver development, and those phenotypes could be partially rescued by corresponding miRNA duplex [75]. Besides these nine key miRNAs mentioned above and two new miRNAs which are not reported in the literature, the rest eight miRNAs also played roles in development, cell proliferation, cell cycle and tumorigenesis [76–78].

Key miRNAs identified in this study regulating a broad spectrum of genes involved in a variety of cellular processes, including development, cell proliferation, bone metabolism, immunity, tumorigenesis, and tissue regeneration, were thus considered to be related to the continuous development and perfection of various tissues in the head (such as brain, eye, mouth and other tissues) and the formation and mineralization of head bone system (cranial skeleton and pharyngeal bone). Further functional characterization of key genes and miRNAs determined by using over expression, knockout and knockdown strategies may help elaborate the molecular mechanisms for head development of bighead carp.

## Materials and methods

### Ethics statement

All experimental protocols involved in fishes in this study were conducted in strict accordance with the recommendations in the Guide for the Care and Use of Laboratory Animals of the Institute of Hydrobiology and ARRIVE guidelines (https://arriveguidelines.org). This study was also approved by the Ethics Committee of Experimental Animals of the Hubei Provincial Committee for Animal Welfare. All efforts were made to minimize suffering of the fish.

### Morphological observation and Fish sample collection

Fish samples used in this study originated from one mixed family of bighead carp, which were cultured at the Zhangdu Lake Fish Farm (Wuhan, China). In May 2019, the offspring were collected at the following developmental stages, including 1Dph (days post hatch) group, 3Dph group, 5Dph group, 7Dph group, 10Dph group, 15Dph group, 20Dph group, 30Dph group and 50Dph group. The total length (TL), body length (BL), and head length (HL) of 15 bighead carp individuals in each development stage were measured in this study. The ratios of HL and BL (HL/BL) from these different development stages were calculated simultaneously. Based on morphological characterizations and the HL/BL values, the whole head in samples of five different developmental stages (1Dph, 3Dph, 5Dph, 15Dph and 30Dph) were selected for the integrated analysis of RNA-seq and sRNA-seq in bighead carp. After anesthetizing, the head of bighead carp from 1Dph, 3Dph and 5Dph were dissected by needles under a dissecting microscope (Zeiss Stemi 2000-C), while the head from 15Dph and 30Dph were dissected by scissors and tweezers. All head tissues (1Dph, 3Dph, 5Dph, 15 Dph and 30Dph) were sampled, quickly frozen in liquid nitrogen and stored at −80 °C prior to RNA extraction.

### Total RNA extraction

Total RNA of mixed samples of head tissues during different developmental stages was extracted using TRIzol Reagent (Invitrogen, CA, USA) referring to a previous report [79]. The RNA degradation and concentration were first detected by 1% agarose gel electrophoresis and NanoDrop 2000 Spectrophotometer (Thermo Scientific, Wilmington, DE, USA), respectively. Then RNA quality was checked using the Agilent Bioanalyzer 2100 system (Agilent Technologies, Santa Clara, CA, USA) and only those samples with the RNA Integrity Number (RIN) value ≥8.0 were used for RNA library construction.

### Transcriptome sequencing and analysis

Fifteen head RNA-Seq libraries from three each of mixed samples during five different developmental stages (1Dph, 3Dph, 5Dph, 15 Dph and 30Dph) were constructed using NEBNext®Ultra™RNA Library Prep Kit for Illumina® (NEB, Ipswich, MA, USA) according to manufacturer’s recommendations. Then libraries were subjected to paired-end sequencing of 150 bp on the Illumina HiSeq Xten platform by Novogene Company (Beijing, China). Before assembly, raw sequencing reads were clipped by removing adapter sequences and ambiguous nucleotides. Then all clean reads of the libraries of head tissues from five different development stages were assembled into transcripts by Trinity software, respectively. After assembly, the TGICL clustering software (J. Craig Venter Institute, Rockville, MD, USA) was used to cluster and remove redundant transcripts, and then the remaining sequences were defined as unigenes. All assembled sequences were used for BLAST searches and annotation against the NCBI non-redundant (Nr) protein, Swiss-Prot, gene ontology (GO), clusters of orthologous groups (COG), euKaryotic orthologous groups (KOG), eggNOG, and protein family (Pfam) databases using an e-value of 1e-5, and the Kyoto Encyclopedia of Genes and Genomes (KEGG) orthology results were obtained by comparing with KEGG database using KOBAS 2.0 [80]. After predicting the amino acid sequence of unigenes, the software HMMER [81] was used to compare them with Pfam database to get the annotation information of unigenes.

Gene expression levels were estimated by RSEM (RNA-Seq by Expectation Maximization) software package using a generative model of RNA-Seq reads and the EM algorithm for each sample [82]. Read count statistics of genes were shown in Table S3. The mapped reads were normalized according to fragment per kilobase of exon model per million mapped reads (FPKM) for each unigene between different development groups. Differentially expressed genes (DEGs) between the two groups were identified by the DEseq2 package (samples with three biological replicates) [83]. The false discovery rate (FDR < 0.01) adjusted by Benjamini–Hochberg method was adopted to get DEGs. DEGs were defined as by parameters of FDR < 0.01 and the absolute value of the log2 ratio ≥ 1. FPKM of DEGs from RNA-Seq was shown in Table S4. In order to determine the potential functions and metabolic pathways of these DEGs, DEGs were annotated against the Nr protein, Swiss-Prot, GO, COG, KOG, eggNOG, Pfam, and KEGG databases. KOBAS 2.0 software [80] was used to performed the statistical enrichment of DEGs in KEGG pathways. The principal component analysis (PCA) and heatmap analysis were conducted by R (Version 3.0.3) ggplot2 package and pheatmap package, respctively.

### Small RNA library sequencing and analysis

Fifteen head sRNA-Seq libraries from three each of mixed samples during five different developmental stages (1Dph, 3Dph, 5Dph, 15 Dph and 30Dph) were generated using NEBNext® Multiplex Small RNA Library Prep Set for Illumina® (NEB, USA.) following manufacturer’s recommendations. Then the fifteen libraries were sequenced on an Illumina Hiseq 2500 platform and 50 bp single-end reads were generated. Raw data of fastq format were firstly processed through custom perl and python scripts to obtain clean data. The clean sequence reads were mapped with miRBase 21.0, allowing a mismatch of one or two nucleotide bases, to identify the known miRNAs. The potential miRNAs and secondary structures were obtained by srna-tools-cli with “srna-tools.pl --tool hp_tool --longSeq --shortSeqs --out” parameter [84]. The available software miREvo [85] and mirdeep2 [86] were integrated to predict novel miRNA through exploring the secondary structure, the Dicer cleavage site and the minimum free energy of the small RNA tags unannotated in the former steps. Hairpin and mature sequences of novel miRNAs from sRNA-Seq was shown in Table S5. Predicting the target gene of miRNA was performed by miRanda [87]. Read count statistics of miRNAs were shown in Table S6. The expression levels of the identified miRNAs were calculated and normalized to TPM (tags per million). Differential expression analysis of the miRNAs was performed using the DESeq R package (v1.8.3) with default parameters, and miRNAs with a corrected P value (p-adjust/padj) < 0.05 were considered as differential expressed miRNAs (DEmiRs). TPM of DEmiRs from sRNA-Seq was shown in Table S7. In order to determine the potential functions and metabolic pathways of the target gene candidates of differentially expressed miRNAs, GOseq [88] and KOBAS [89] were implemented for GO enrichment analysis and KEGG pathway enrichment analysis of the target gene candidates of differentially expressed miRNAs.

### Quantitative PCR for miRNA and mRNA expression

In order to examine the reliability of the RNA-Seq and the sRNA-Seq results, 6 DEGs and 6 DEmiRs were randomly selected for validation by qRT-PCR method. Total RNA from 15 samples (three each of five different developmental stages) was extracted individually using TRIzol Reagent (Invitrogen, CA, USA) according to the manufacturer’s instruction. 1 μg of total RNA for each sample was used to synthesize cDNA by using PrimeScript™ RT reagent Kit (TaKaRa, Dalian, China) for DEGs and miRNA first-strand cDNA synthesis kit (Vazyme, Nanjing, China) with stem-loop reverse transcription primers for DEmiRs according to the manufacturer’s protocols, respectively. Compared with stem-loop reverse transcription primers of DEmiRs, the reverse transcription primers of U6 reference gene can be directly designed by conventional primer premier 5 software. The downstream primer of U6 can be input to make reverse transcription according to the manufacturer’s protocols (Vazyme, Nanjing, China). The PCR primers for DEGs and DEmiRs were list in Table S8. The qRT-PCR reaction solution was 20 μL, containing 10 μL of SYBR Green PCR Master Mix (TaKaRa, Dalian, China), 3 μL of forward and reverse primers (2 μmol/L), 2 μL of cDNA (500 ng) and 2 μL of sterile distilled water. qRT-PCR amplification was performed on a StepOne™ Real-Time PCR System (Applied Biosystems, USA) according to a previous report [79]. RNA samples of head tissues from five different developmental stages were run in three biological replicates and three technical replicates for qRT-PCR. The expression level of each DEG and DEmiR was normalized to that of the reference gene *β-actin* and *U6* by using the 2^−ΔΔCT^ method [90] to validate the results of RNA-seq and sRNA-seq, respectively.

## Conclusions

This study represents the first application of RNA–Seq and sRNA–Seq in conducting integrated analyses for the head of bighead carp during different development stages, including 1Dph, 3Dph, 5Dph, 15Dph and 30Dph. Firstly, 26 pathways related to head development and bone formation were enriched and identified as the main pathways during early growth. Coupling this to sRNA–Seq data, we picked out six key pathways that may be responsible for head development, namely ECM receptor interaction, TNF signaling pathway, osteoclast differentiation, PI3K–Akt signaling pathway, Neuroactive ligand–receptor interaction and Jak–STAT signaling pathway. Totally, 114 important candidate genes were obtained from these six pathways. Then top 20 key genes were identified according to the degree value by cytohubba, which regulated cell growth, skeletal development and blood homeostasis, such as *pik3ca*, *pik3r1*, *egfr*, *vegfa*, *igf1* and *itga2b*. Finally, 19 key miRNAs playing multiple roles in the perfection of various tissues in the head (such as brain, eye, muscle, lipids and mouth) and mineralization of head bone system, such as let–7e, miR–142a–5p, miR–144–3p, miR–23a–3p and miR–223 were also acquired. Results of this study will shed lights on further understanding of genetic mechanisms underlying head development and also provide potential candidate targets for the interaction regulation during early growth in bighead carp.

## Supplementary Information

**Additional file 1: Table S1**. Summary statistics for sequencing information of the bighead carp head transcriptome.

**Additional file 2: Table S2**. Annotation information of uingenes from RNA-Seq. (XLS 48557 kb)

**Additional file 3: Table S3**. Read count statistics of genes from RNA-Seq.

**Additional file 4: Table S4**. FPKM of differential expressed genes from RNA-Seq.

**Additional file 5: Table S5**. Hairpin and mature sequences of novel miRNAs from sRNA-Seq.

**Additional file 6: Table S6**. Read count statistics of miRNAs from sRNA-Seq.

**Additional file 7: Table S7**. TPM of differential expressed miRNAs from sRNA-Seq.

**Additional file 8: Table S8**. Primer sequences of the DEGs and DEmiRs for qRT-PCR.

**Additional file 9: Figure S1**. The size distribution of the unigenes from head tissues in bighead carp.

**Additional file 10: Figure S2**. Key genes related to head development in bighead carp. The FPKM data of genes was used for heatmap construction. Gene abbreviations: Epidermal growth factor receptor (*EGFR*), Prothrombin (*F2*), Proteinase-activated receptor 1 (*F2R*), Fibronectin type III domain containing (*FN1*), Glucagon receptor (*GCGR*), Hepatocyte growth factor (*HGF*), Insulin-like growth factor I (*IGF1*), Integrin alpha-IIb (*ITGA2B*), Integrin beta-3 (*ITGB3*), Tyrosine-protein kinase JAK1 (*JAK1*), Vascular endothelial growth factor receptor 2 (*KDR*), Mast/stem cell growth factor receptor Kit (*KIT*), Platelet-derived growth factor receptor beta (*PDGFRB*), Phosphatidylinositol 4,5-bisphosphate 3-kinase catalytic subunit alpha isoform (*PIK3CA*), Phosphatidylinositol 3-kinase regulatory subunit alpha (*PIK3R1*), Signal transducer and activator of transcription 1-alpha/beta (*STAT1*), Signal transducer and activator of transcription 3 (*STAT3*), Thrombospondin-1 (*THBS1*), Vascular endothelial growth factor A (*VEGFA*), Von Willebrand factor (*VWF*).

**Additional file 11: Figure S3**. qRT-PCR validation of DEGs and DEmiRs of head tissues in bighead carp during different development stages.

## Data Availability

All raw reads are available in the Sequence Read Archive of National Center for Biotechnology Information database with accession number PRJNA731038 (https://www.ncbi.nlm.nih.gov/search/all/?term=PRJNA731038). Please contact Weiwei Luo (weiweiluo66@163.com) if someone wants to request the data from this study.

## References

[1] Depew MJ, Olsson L (2008). Symposium on the evolution and development of the vertebrate head. J Exp Zool Part B.

[2] Geng X, Liu S, Yao J, Bao L, Zhang J, Li C (2016). A Genome-Wide Association Study Identifies Multiple Regions Associated with Head Size in Catfish. G3-Genes Genom Genet.

[3] Sutter NB, Bustamante CD, Chase K, Gray MM, Zhao K, Zhu L (2007). A single IGF1 allele is a major determinant of small size in dogs. Science..

[4] Schoenebeck JJ, Ostrander EA (2013). The Genetics of Canine Skull Shape Variation. Genetics..

[5] Schneider RA, Helms JA (2003). The cellular and molecular origins of beak morphology. Science..

[6] Duhamel A, Hume JP, Guenser P, Salaviale C, Louchart A (2020). Cranial evolution in the extinct Rodrigues Island owl *Otus murivorus* (Strigidae), associated with unexpected ecological adaptations. Sci Rep-UK..

[7] Diogo R, Razmadze D, Siomava N, Douglas N, Fuentes JSM, Duerinckx A. Musculoskeletal study of cebocephalic and cyclopic lamb heads illuminates links between normal and abnormal development, evolution and human pathologies. Sci Rep-UK. 2019;9:991. 10.1038/s41598-018-37735-9 PMC6353885 30700788

[8] Wang Y, Zhang C, Wang N, Li Z, Heller R, Liu R (2019). Genetic basis of ruminant headgear and rapid antler regeneration. Science..

[9] Rutten MJM, Bovenhuis H, Komen H (2005). Genetic parameters for fillet traits and body measurements in Nile tilapia (*Oreochromis niloticus* L.). Aquaculture..

[10] Chen L, Peng W, Kong S, Pu F, Chen B, Zhou Z, et al. Genetic Mapping of Head Size Related Traits in Common Carp (*Cyprinus carpio*). Front Genet. 2018;9:448. 10.3389/fgene.2018.00448 PMC6190898 30356829

[11] Liu JH, Zhang Y, Chang YM, Liang LQ, Lu CY, Zhang XF (2009). Mapping QTLs related to head length, eye diameter and eye cross of common carp (*Cyprinus carpio* L.). Yi chuan. Hereditas..

[12] Jin S, Zhang X, Jia Z, Fu H, Zheng X, Sun X (2012). Genetic linkage mapping and genetic analysis of QTL related to eye cross and eye diameter in common carp (*Cyprinus carpio* L.) using microsatellites and SNPs. Aquaculture..

[13] Szabo T, Urbanyi B, Muller T, Szabo R, Horvath L. Assessment of induced breeding of major Chinese carps at a large-scale hatchery in Hungary. Aquacult Rep. 2019;14:100193.

[14] Hong H, Luo Y, Zhou Z, Bao Y, Lu H, Shen H (2013). Effects of different freezing treatments on the biogenic amine and quality changes of bighead carp (*Aristichthys nobilis*) heads during ice storage. Food Chem.

[15] Ambros V (2004). The functions of animal microRNAs. Nature..

[16] Krol J, Loedige I, Filipowicz W (2010). The widespread regulation of microRNA biogenesis, function and decay. Nat Rev Genet.

[17] Cheng AM, Byrom MW, Shelton J, Ford LP (2005). Antisense inhibition of human miRNAs and indications for an involvement of miRNA in cell growth and apoptosis. Nucleic Acids Res.

[18] Wan SM, Yi SK, Zhong J, Nie CH, Guan NN, Zhang WZ, et al. Dynamic mRNA and miRNA expression analysis in response to intermuscular bone development of blunt snout bream (*Megalobrama amblycephala*). Sci Rep-UK. 2016;6:31050. 10.1038/srep31050 PMC4971466 27486015

[19] Ji C, Guo X, Ren J, Zu Y, Li W, Zhang Q (2019). Transcriptomic analysis of microRNAs-mRNAs regulating innate immune response of zebrafish larvae against Vibrio parahaemolyticus infection. Fish Shellfish Immun..

[20] Li S, Lin G, Fang W, Gao D, Huang J, Xie J, et al. Identification and Comparison of microRNAs in the Gonad of the Yellowfin Seabream (*Acanthopagrus Latus*). Int J Mol Sci. 2020;21(16):5690. 10.3390/ijms21165690 PMC7461063 32784462

[21] Ning X, Sun L. Micro-Transcriptome Analysis Reveals Immune-Related MicroRNA Regulatory Networks of *Paralichthys olivaceus* Induced by *Vibrio anguillarum* Infection. Int J Mol Sci. 2020;21(12):4252. 10.3390/ijms21124252 PMC7352997 32549342

[22] Fuller-Carter PI, Carter KW, Anderson D, Harvey AR, Giles KM, Rodger J. Integrated analyses of zebrafish miRNA and mRNA expression profiles identify miR-29b and miR-223 as potential regulators of optic nerve regeneration. Bmc. Genomics. 2015;16:591. 10.1186/s12864-015-1772-1 PMC4534052 26265132

[23] Zhang X, Wang G, Sun Z, Hou J, Wang Y (2019). microRNA-mRNA analysis in pituitary and hypothalamus of sterile Japanese flounder. Mol Reprod Dev.

[24] Sun JL, Zhao LL, He K, Liu Q, Luo J, Zhang DM, et al. MiRNA-mRNA integration analysis reveals the regulatory roles of miRNAs in the metabolism of largemouth bass (*Micropterus salmoides* ) livers during acute hypoxic stress. Aquaculture. 2020;526:735362.

[25] Nie CH, Wan SM, Liu YL, Liu H, Wang WM, Gao ZX. Development of Teleost Intermuscular Bones Undergoing Intramembranous Ossification Based on Histological-Transcriptomic-Proteomic Data. Int J Mol Sci. 2019;20(19):4698. 10.3390/ijms20194698 PMC6801895 31546739

[26] Kong S, Zhou Z, Zhou T, Zhao J, Chen L, Lin H (2020). Genome-Wide Association Study of Body Shape-Related Traits in Large Yellow Croaker (*Larimichthys crocea*). Mar Biotechnol.

[27] Wu P, Zhang X, Zhang G, Chen F, He M, Zhang T, et al. Transcriptome for the breast muscle of Jinghai yellow chicken at early growth stages. Peerj. 2020;8:e8950. 10.7717/peerj.8950 PMC7166044 32328350

[28] Wang Y, Gao W (2021). Effects of TNF-alpha on autophagy of rheumatoid arthritis fibroblast-like synoviocytes and regulation of the NF-kappaB signaling pathway. Immunobiology..

[29] Hurem S, Martin LM, Brede DA, Skjerve E, Nourizadeh-Lillabadi R, Lind OC, et al. Dose-dependent effects of gamma radiation on the early zebrafish development and gene expression. PLoS One. 2017;12(6):e0179259. 10.1371/journal.pone.0179259 PMC5476279 28628668

[30] Boyle WJ, Simonet WS, Lacey DL (2003). Osteoclast differentiation and activation. Nature..

[31] Tu Y, Liu Y, Zhang M, Shan Y, Ji G, Ju X (2021). Identifying Signatures of Selection Related to Comb Development. J Poult Sci.

[32] Zhang Y, Jiao Y, Li Y, Tian Q, Du X, Deng Y. Comprehensive analysis of microRNAs in the mantle central and mantle edge provide insights into shell formation in pearl oyster *Pinctada fucata martensii*. Comp Biochem Phys B. 2021;252:110508. 10.1016/j.cbpb.2020.110508 32992005

[33] Harrison DA (2012). The JAK/STAT Pathway. CSH Perspect Biol.

[34] Meier TI, Cook JA, Thomas JE, Radding JA, Horn C, Lingaraj T (2004). Cloning, expression, purification, and characterization of the human Class Ia phosphoinositide 3-kinase isoforms. Protein Expres Purif.

[35] Miled N, Yan Y, Hon W-C, Perisic O, Zvelebil M, Inbar Y (2007). Mechanism of two classes of cancer mutations in the phosphoinositide 3-kinase catalytic subunit. Science..

[36] Bi L, Okabe I, Bernard DJ, Wynshaw-Boris A, Nussbaum RL (1999). Proliferative defect and embryonic lethality in mice homozygous for a deletion in the p110 alpha subunit of phosphoinositide 3-kinase. J Biol Chem.

[37] Runkle KB, Kharbanda A, Stypulkowski E, Cao XJ, Wang W, Garcia BA (2016). Inhibition of DHHC20-Mediated EGFR Palmitoylation Creates a Dependence on EGFR Signaling. Mol Cell.

[38] Zoidis E, Ghirlanda-Keller C, Schmid C (2011). Stimulation of glucose transport in osteoblastic cells by parathyroid hormone and insulin-like growth factor I. Mol Cell Biochem.

[39] Tolbert WD, Daugherty-Holtrop J, Gherardi E, Woude GV, Xu HE (2010). Structural basis for agonism and antagonism of hepatocyte growth factor. P Natl Acad Sci USA.

[40] Liu JP, Baker J, Perkins AS, Robertson EJ, Efstratiadis A (1993). mice carrying null mutations of the genes encoding insulin-like growth factor-i (igf-1) and type-1 igf receptor (igf1r). Cell..

[41] Romero CJ, Ng Y, Luque RM, Kineman RD, Koch L, Bruning JC (2010). Targeted Deletion of Somatotroph Insulin-Like Growth Factor-I Signaling in a Cell-Specific Knockout Mouse Model. Mol Endocrinol.

[42] Chaix A, Lopez S, Voisset E, Gros L, Dubreuil P, De Sepulveda P (2011). Mechanisms of STAT Protein Activation by Oncogenic KIT Mutants in Neoplastic Mast Cells. J Biol Chem.

[43] Owens RJ, Baralle FE (1986). Mapping the collagen-binding site of human fibronectin by expression in escherichia-coli. EMBO J.

[44] Brunner M, Millon-Fremillon A, Chevalier G, Nakchbandi IA, Mosher D, Block MR (2011). Osteoblast mineralization requires beta 1 integrin/ICAP-1-dependent fibronectin deposition. J Cell Biol.

[45] Kaplan N, Yilmaz I, Karaarslan N, Kaya YE, Sirin DY, Ozbek H (2019). Does Nimodipine, a Selective Calcium Channel Blocker, Impair Chondrocyte Proliferation or Damage Extracellular Matrix Structures?. Curr Pharm Biotechno.

[46] Shao H, Cheng HYY, Cook RG, Tweardy DJ (2003). Identification and characterization of signal transducer and activator of transcription 3 recruitment sites within the epidermal growth factor receptor. Cancer Res.

[47] Saxena NK, Vertino PM, Anania FA, Sharma D (2007). Leptin-induced growth stimulation of breast cancer cells involves recruitment of histone acetyltransferases and mediator complex to CYCLIN D1 promoter via activation of Stat3. J Biol Chem.

[48] Simantov R, Febbraio M, Crombie R, Asch AS, Nachman RL, Silverstein RL (2001). Histidine-rich glycoprotein inhibits the antiangiogenic effect of thrombospondin-1. J Clin Invest.

[49] Murphy JF, Fitzgerald DJ (2001). Vascular endothelial cell growth factor (VEGF) induces cyclooxygenase (COX)-dependent proliferation of endothelial cells (EC) via the VEGF-2 receptor. FASEB J.

[50] Terman BI, Doughervermazen M, Carrion ME, Dimitrov D, Armellino DC, Gospodarowicz D (1992). identification of the kdr tyrosine kinase as a receptor for vascular endothelial-cell growth-factor. Biochem Bioph Res Co.

[51] Rho SB, Song YJ, Lim MC, Lee S-H, Kim B-R, Park S-Y (2012). Programmed cell death 6 (PDCD6) inhibits angiogenesis through PI3K/mTOR/p70S6K pathway by interacting of VEGFR-2. Cellular Signal.

[52] Glenn KC, Frost GH, Bergmann JS, Carney DH (1988). Synthetic peptides bind to high-affinity thrombin receptors and modulate thrombin mitogenesis. J Pept Res.

[53] Kahn ML, Nakanishi-Matsui M, Shapiro MJ, Ishihara H, Coughlin SR (1999). Protease-activated receptors 1 and 4 mediate activation of human platelets by thrombin. J Clin Invest.

[54] Yin Q, Gu J, Qi Y, Lu Y, Yang L, Liu J, et al. Adam28 from both endothelium and gastric cancer cleaves von Willebrand Factor to eliminate von Willebrand Factor-induced apoptosis of gastric cancer cells. Eur J Pharmacol. 2021;898:173994. 10.1016/j.ejphar.2021.173994 33675784

[55] Sakatsume M, Igarashi K, Winestock KD, Garotta G, Larner AC, Finbloom DS (1995). The jak kinases differentially associate with the alpha and beta (accessory factor) chains of the interferon-gamma receptor to form a functional receptor unit capable of activating stat transcription factors. J Biol Chem.

[56] Koth CM, Murray JM, Mukund S, Madjidi A, Minn A, Clarke HJ (2012). Molecular basis for negative regulation of the glucagon receptor. P Natl Acad Sci USA..

[57] Kim HJ, Cha B-Y, Choi B, Lim JS, Woo JT, Kim JS (2012). Glyceollins inhibit platelet-derived growth factor-mediated human arterial smooth muscle cell proliferation and migration. Brit J Nutr.

[58] Li Z, Pan W, Shen Y, Chen Z, Zhang L, Zhang Y (2018). IGF1/IGF1R and microRNA let-7e down-regulate each other and modulate proliferation and migration of colorectal cancer cells. Cell Cycle.

[59] Zhang Y, Cheng W, Han B, Guo Y, Wei S, Yu L (2021). Let-7i-5p functions as a putative osteogenic differentiation promoter by targeting CKIP-1. Cytotechnology..

[60] Hu P, Wang T, Liu H, Xu J, Wang L, Zhao P (2019). Full-length transcriptome and microRNA sequencing reveal the specific gene-regulation network of velvet antler in sika deer with extremely different velvet antler weight. Mol Gen Genomics.

[61] Yuan X, Berg N, Lee JW, Thanh-Thuy L, Neudecker V, Jing N (2018). MicroRNA miR-223 as regulator of innate immunity. J Leukoc Biol.

[62] Xie Y, Zhang L, Gao Y, Ge W, Tang P (2015). The Multiple Roles of Microrna-223 in Regulating Bone Metabolism. Molecules..

[63] Sun JL, Zhao LL, He K, Liu Q, Luo J, Zhang DM (2020). MicroRNA regulation in hypoxic environments: differential expression of microRNAs in the liver of largemouth bass (*Micropterus salmoides*). Fish Physiol Biochem.

[64] Dai Y, Zheng C, Li H. Inhibition of miR-23a-3p promotes osteoblast proliferation and differentiation. J Cell Biochem. 2019;122(6):915-26. 10.1002/jcb.29497 31691358

[65] Hu H, Dong L, Bu Z, Shen Y, Luo J, Zhang H, et al. miR-23a-3p-abundant small extracellular vesicles released from Gelma/nanoclay hydrogel for cartilage regeneration. J Extracell Vesicles. 2020;9(1):1778883. 10.1080/20013078.2020.1778883 PMC7480606 32939233

[66] Fang Y, Xu XY, Shen Y, Li J (2020). miR-23a-3p and miR-23a-5p target CiGadd45ab to modulate inflammatory response and apoptosis in grass carp. Fish Shellfish Immun..

[67] Yang X, Yang S, Song J, Yang W, Ji Y, Zhang F, et al. Dysregulation of miR-23b-5p promotes cell proliferation via targeting FOXM1 in hepatocellular carcinoma. Cell Death Discov. 2021;7(1):47. 10.1038/s41420-021-00440-0 PMC7960996 33723252

[68] You L, Wang Y, Gao Y, Wang X, Cui X, Zhang Y (2020). The role of microRNA-23b-5p in regulating brown adipogenesis and thermogenic program. Endocr Connect.

[69] Fan K, Shen Y, Xu X, Tao L, Bao T, Li J (2021). LncRNA-WAS and lncRNA-C8807 interact with miR-142a-3p to regulate the inflammatory response in grass carp. Fish Shellfish Immun.

[70] Lalwani MK, Sharma M, Singh AR, Chauhan RK, Patowary A, Singh N, et al. Reverse Genetics Screen in Zebrafish Identifies a Role of miR-142a-3p in Vascular Development and Integrity. PLoS One. 2012;7(12):e52588. 10.1371/journal.pone.0052588 PMC3528674 23285103

[71] Woldemariam NT, Agafonov O, Sindre H, Hoyheim B, Houston RD, Robledo D, et al. miRNAs Predicted to Regulate Host Anti-viral Gene Pathways in IPNV-Challenged Atlantic Salmon Fry Are Affected by Viral Load, and Associated With the Major IPN Resistance QTL Genotypes in Late Infection. Front Immuno. 2020;11:2113. 10.3389/fimmu.2020.02113 PMC7516080 33013890

[72] Eslamloo K, Inkpen SM, Rise ML, Andreassen R (2018). Discovery of microRNAs associated with the antiviral immune response of Atlantic cod macrophages. Mol Immunol.

[73] Huang CX, Chen N, Wu XJ, Huang CH, He Y, Tang R (2015). The zebrafish miR-462/miR-731 cluster is induced under hypoxic stress via hypoxia-inducible factor 1 alpha and functions in cellular adaptations. FASEB J.

[74] Huang CH, Chen N, Huang CX, Zhang B, Wu M, He L (2017). Involvement of the miR-462/731 cluster in hypoxia response in *Megalobrama amblycephala*. Fish Physiol Biochem.

[75] Huang Y, Huang CX, Wang WF, Liu H, Wang HL. Zebrafish miR-462-731 is required for digestive organ development. Comp Biochem Phys D. 2020;34:100679. 10.1016/j.cbd.2020.100679 32200130

[76] Wu P, Shi J, Yang C, Zhang F, Li Y, Chen L (2018). Effects of short-term starvation on the rhythmic expression of microRNAs in skeletal muscle of goldfish (*Carassius auratus*). Aquac Res.

[77] Khordadmehr M, Jigari-Asl F, Ezzati H, Shahbazi R, Sadreddini S, Safaei S (2019). A comprehensive review on miR-451: A promising cancer biomarker with therapeutic potential. J Cell Physiol.

[78] Li N, Liu L, Liu Y, Luo S, Song Y, Fang B (2020). miR-144-3p Suppresses Osteogenic Differentiation of BMSCs from Patients with Aplastic Anemia through Repression of TET2. Mol Ther-Nucl Acids.

[79] Zhou Y, Luo WW, Yu XM, Liu QS, Tong JG (2019). Brain and intestine transcriptome analyses and identification of genes involved in feed conversion efficiency of Yellow River carp (*Cyprinus carpio haematopterus*). Comp Biochem Phys D.

[80] Xie C, Mao X, Huang J, Ding Y, Wu J, Dong S (2011). KOBAS 2.0: a web server for annotation and identification of enriched pathways and diseases. Nucleic Acids Res.

[81] Eddy SR (1998). Profile hidden Markov models. Bioinformatics..

[82] Li B, Dewey CN. RSEM: accurate transcript quantification from RNA-Seq data with or without a reference genome. Bmc. Bioinformatics. 2011;12(1):323. 10.1186/1471-2105-12-323 PMC3163565 21816040

[83] Anders S, Huber W. Differential expression analysis for sequence count data. Genome Biol. 2010;11(10):R106. 10.1186/gb-2010-11-10-r106 PMC3218662 20979621

[84] Moxon S, Schwach F, Dalmay T, Maclean D, Studholme DJ, Moulton V (2008). A toolkit for analysing large-scale plant small RNA datasets. Bioinformatics..

[85] Wen M, Shen Y, Shi S, Tang T. miREvo: an integrative microRNA evolutionary analysis platform for next-generation sequencing experiments. Bmc. Bioinformatics. 2012;13:140. 10.1186/1471-2105-13-140 PMC3410788 22720726

[86] Friedlaender MR, Mackowiak SD, Li N, Chen W, Rajewsky N (2012). miRDeep2 accurately identifies known and hundreds of novel microRNA genes in seven animal clades. Nucleic Acids Res.

[87] Enright AJ, John B, Gaul U, Tuschl T, Sander C, Marks DS (2004). MicroRNA targets in Drosophila. Genome Biol.

[88] Young MD, Wakefield MJ, Smyth GK, Oshlack A. Gene ontology analysis for RNA-seq: accounting for selection bias. Genome Biol. 2010;11(2):R14. 10.1186/gb-2010-11-2-r14 PMC2872874 20132535

[89] Mao XZ, Cai T, Olyarchuk JG, Wei LP (2005). Automated genome annotation and pathway identification using the KEGG Orthology (KO) as a controlled vocabulary. Bioinformatics..

[90] Livak KJ, Schmittgen TD (2001). Analysis of relative gene expression data using real-time quantitative PCR and the 2(T)(−Delta Delta C) method. Methods..

