# Supplementary material for: Dynamic mRNA and miRNA expression of the head during early development in bighead carp (Hypophthalmichthys nobilis)
Source: BMC Genomics. 2022 Mar 1;23:168. doi: 10.1186/s12864-022-08387-x (PMC8887032; doi:10.1186/s12864-022-08387-x)
Supplement: Supplementary file 1 — Additional file 1: Table S1. Summary statistics for sequencing information of the bighead carp head transcriptome. [file 12864_2022_8387_MOESM1_ESM.docx]

**Supplementary Table S1** Summary statistics for sequencing information of the bighead carp head transcriptome

| **Item** | **Sample** | **Clean Reads** | **Clean Bases** | **GC Content** | **%≥Q30** |
| --- | --- | --- | --- | --- | --- |
| Dph1 | Dph1-1 | 61295948 | 9.19G | 46.26% | 94.48% |
|  | Dph1-2 | 52481148 | 7.87G | 46.07% | 94.43% |
|  | Dph1-3 | 69810818 | 10.47G | 46.66% | 94.38% |
| Dph3 | Dph3-1 | 54147804 | 8.12G | 46.00% | 94.60% |
|  | Dph3-2 | 67985780 | 10.2G | 46.48% | 94.68% |
|  | Dph3-2 | 56877450 | 8.53G | 45.89% | 94.43% |
| Dph5 | Dph5-1 | 55183012 | 8.28G | 46.12% | 94.35% |
|  | Dph5-2 | 53148002 | 7.97G | 46.41% | 94.35% |
|  | Dph5-3 | 69013958 | 10.35G | 45.99% | 94.55% |
| Dph15 | Dph15-1 | 63690934 | 9.55G | 46.49% | 94.69% |
|  | Dph15-2 | 87986564 | 13.2G | 46.49% | 94.59% |
|  | Dph15-3 | 66024564 | 9.9G | 46.42% | 94.84% |
|  | Dph30-1 | 52214166 | 7.83G | 45.83% | 94.53% |
| Dph30 | Dph30-2 | 51037958 | 7.66G | 46.31% | 94.85% |
|  | Dph30-3 | 52338548 | 7.85G | 46.59% | 95.01% |

Dph: days post hatch.

GC% is the percentage of proportion of guanidine and cytosine nucleotides among total nucleotides.

Q30 is the percentage of bases with quality value larger than 30.
