# Supplementary figures and images for: Dynamic mRNA and miRNA expression of the head during early development in bighead carp (Hypophthalmichthys nobilis)
Source: BMC Genomics. 2022 Mar 1;23:168. doi: 10.1186/s12864-022-08387-x (PMC8887032; doi:10.1186/s12864-022-08387-x)

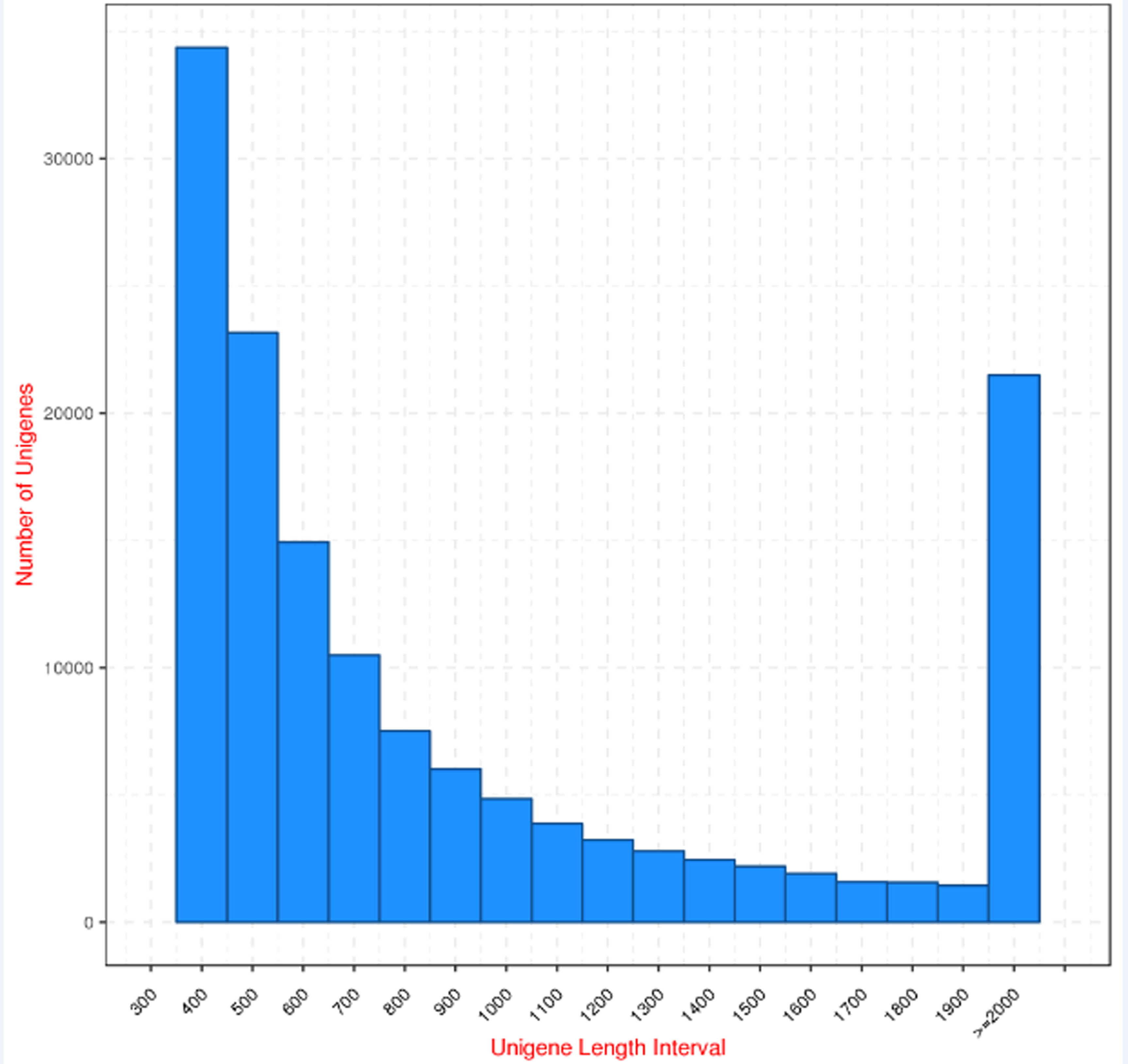

Supplement: Supplementary file 9 — Additional file 9: Figure S1. The size distribution of the unigenes from head tissues in bighead carp. [file 12864_2022_8387_MOESM9_ESM.png]

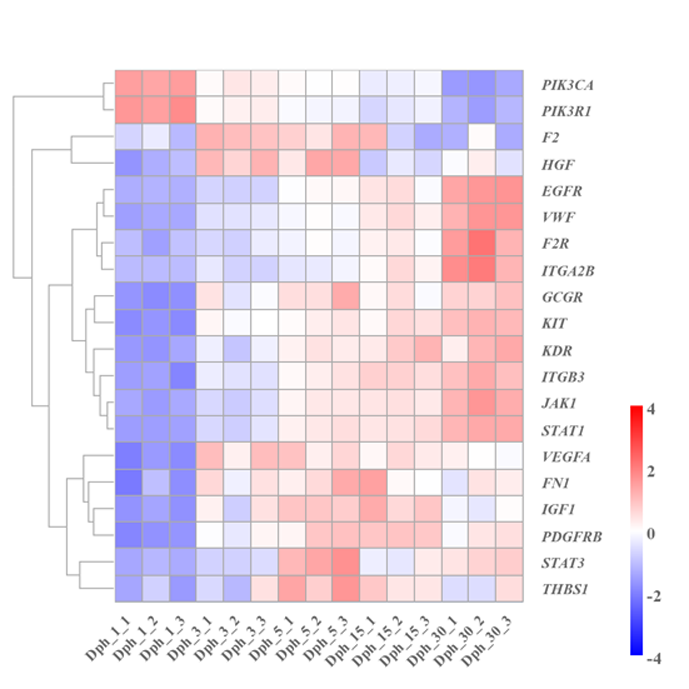

Supplement: Supplementary file 10 — Additional file 10: Figure S2. Key genes related to head development in bighead carp. The FPKM data of genes was used for heatmap construction. Gene abbreviations: Epidermal growth factor receptor (EGFR), Prothrombin (F2), Proteinase-activated receptor 1 (F2R), Fibronectin type III domain containing (FN1), Glucagon receptor (GCGR), Hepatocyte growth factor (HGF), Insulin-like growth factor I (IGF1), Integrin alpha-IIb (ITGA2B), Integrin beta-3 (ITGB3), Tyrosine-protein kinase JAK1 (JAK1), Vascular endothelial growth factor receptor 2 (KDR), Mast/stem cell growth factor receptor Kit (KIT), Platelet-derived growth factor receptor beta (PDGFRB), Phosphatidylinositol 4,5-bisphosphate 3-kinase catalytic subunit alpha isoform (PIK3CA), Phosphatidylinositol 3-kinase regulatory subunit alpha (PIK3R1), Signal transducer and activator of transcription 1-alpha/beta (STAT1), Signal transducer and activator of transcription 3 (STAT3), Thrombospondin-1 (THBS1), Vascular endothelial growth factor A (VEGFA), Von Willebrand factor (VWF). [file 12864_2022_8387_MOESM10_ESM.png]

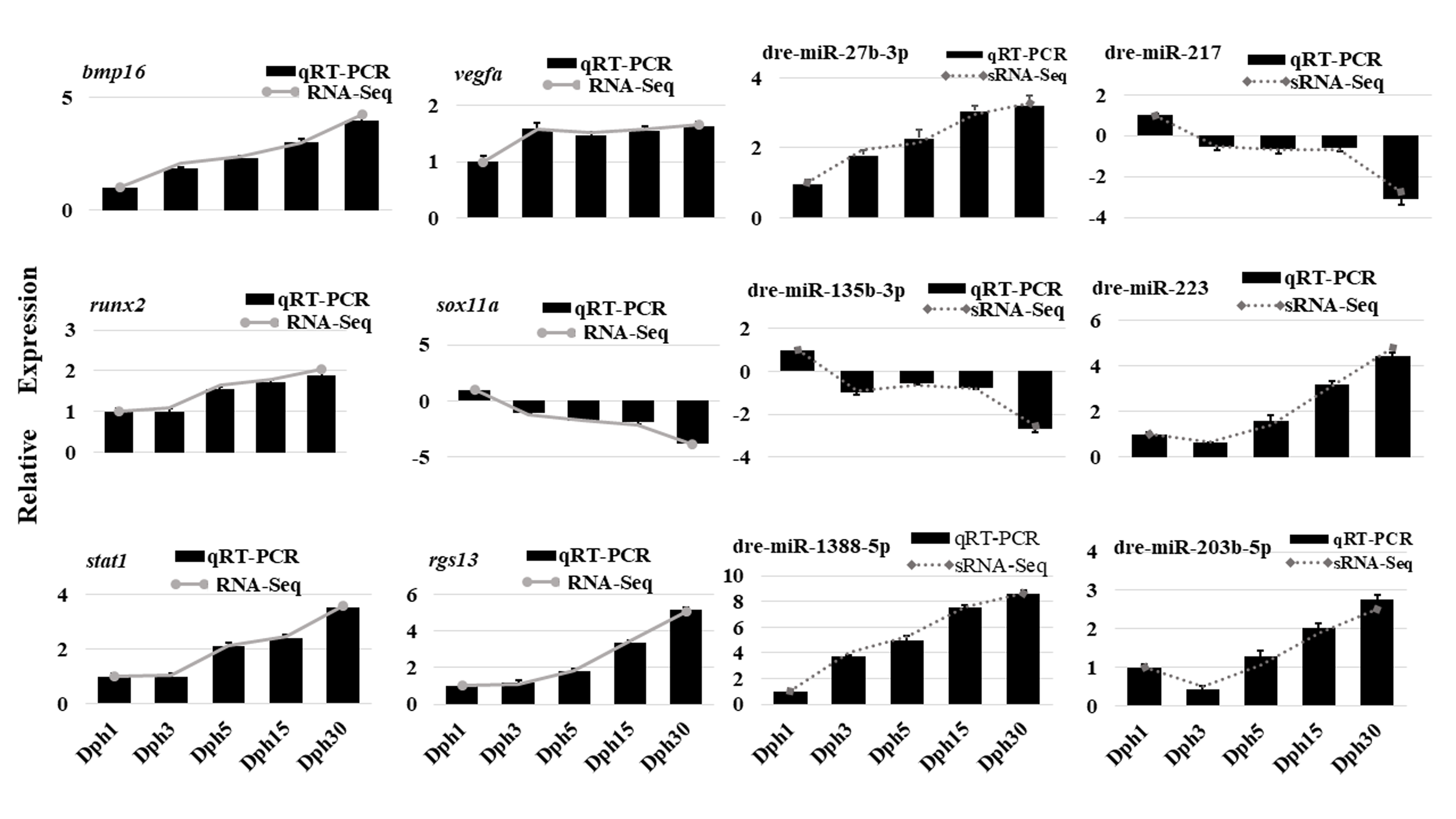

Supplement: Supplementary file 11 — Additional file 11: Figure S3. qRT-PCR validation of DEGs and DEmiRs of head tissues in bighead carp during different development stages. [file 12864_2022_8387_MOESM11_ESM.png]
